# Compared to conventional, ecological intensive management promotes beneficial proteolytic soil microbial communities for agro-ecosystem functioning under climate change-induced rain regimes

**DOI:** 10.1038/s41598-020-64279-8

**Published:** 2020-04-29

**Authors:** Martina Lori, Gabin Piton, Sarah Symanczik, Nicolas Legay, Lijbert Brussaard, Sebastian Jaenicke, Eduardo Nascimento, Filipa Reis, José Paulo Sousa, Paul Mäder, Andreas Gattinger, Jean-Christophe Clément, Arnaud Foulquier

**Affiliations:** 1 Department of Soil Sciences, Research Institute of Organic Agriculture (FiBL), Ackerstrasse 113, 5070 Frick, Switzerland; 2 Organic Farming with focus on Sustainable Soil Use, Karl-Glöckner-Str. 21 C, Justus-Liebig University Giessen, 35394 Giessen, Germany; 3 Univ. Grenoble Alpes, Univ. Savoie Mont Blanc, CNRS, LECA, 38000 Grenoble, France; 4 École de la Nature et du Paysage, INSA Centre Val de Loire, 41000 Blois - CNRS, CITERES, UMR 7324, 37200, Tours, France; 5 Soil Biology Group, Wageningen University & Research, P.O. Box 47, 6700 AA, Wageningen, The Netherlands; 6 Bioinformatics and Systems Biology, Heinrich-Buff-Ring 58, Justus-Liebig-University Giessen, 35392 Giessen, Germany; 7 Centre for Functional Ecology, Department of Life Sciences, University of Coimbra, 3000-456 Coimbra, Portugal; 8 Univ. Savoie Mont Blanc, INRAE, CARRTEL, 74200 Thonon-Les-Bains, France

**Keywords:** Ecology, Agroecology, Climate-change ecology, Ecosystem ecology, Grassland ecology, Microbial ecology, Molecular ecology

## Abstract

Projected climate change and rainfall variability will affect soil microbial communities, biogeochemical cycling and agriculture. Nitrogen (N) is the most limiting nutrient in agroecosystems and its cycling and availability is highly dependent on microbial driven processes. In agroecosystems, hydrolysis of organic nitrogen (N) is an important step in controlling soil N availability. We analyzed the effect of management (ecological intensive *vs*. conventional intensive) on N-cycling processes and involved microbial communities under climate change-induced rain regimes. Terrestrial model ecosystems originating from agroecosystems across Europe were subjected to four different rain regimes for 263 days. Using structural equation modelling we identified direct impacts of rain regimes on N-cycling processes, whereas N-related microbial communities were more resistant. In addition to rain regimes, management indirectly affected N-cycling processes via modifications of N-related microbial community composition. Ecological intensive management promoted a beneficial N-related microbial community composition involved in N-cycling processes under climate change-induced rain regimes. Exploratory analyses identified phosphorus-associated litter properties as possible drivers for the observed management effects on N-related microbial community composition. This work provides novel insights into mechanisms controlling agro-ecosystem functioning under climate change.

## Introduction

As in many terrestrial ecosystems, nitrogen (N) is the most limiting nutrient for plant growth in agroecosystems^1–3^. The last century has been characterized by a considerable increase of N inputs in agricultural soils^4–7^, mostly in mineral form (NH_4_^+^), making plant growth less dependent on microbial N provisioning. However, the increased amount of reactive N in the environment has severe environmental and human health consequences^7^. Ecological intensification has been proposed as an alternative agricultural approach, integrating ecological processes into management strategies of agroecosystems in order to reduce anthropogenic N inputs and enhance ecosystem services^8^. Ecological intensive management (e.g. organic farming) can increase soil organic carbon content^9^, change plant traits^10^, increase soil microbial abundance and activity^11^ and affect diversity as well as select for a distinct microbial community composition compared to conventional intensively managed systems^12^. Consequently, ecological intensive managed soils are more dependent on N-cycling processes driven by microbes^4^. The general importance of microbes in driving soil N mineralization and availability was recently confirmed by a meta-analysis compiling data from nearly two-hundred studies^13^. Several experimental studies have shown a link between microbial diversity and soil processes^14,15^ and evidence showing a tight association between plant traits, soil microbial community properties and ecosystem functioning is accumulating^16,17^. On a global scale, it was recently identified that microbial diversity is positively related to multifunctionality in terrestrial ecosystems^18^. Authors suggest that loss of microbial diversity, likely resulting from human activities and climate change, will result in reduced rates at which multiple ecosystem processes and services are maintained. In addition, it has been demonstrated that an increase in soil biodiversity, including microbial diversity, as well as the promotion of a specific microbial community composition should be part of ecological intensification in agriculture^19^. Soil microbial diversity and composition might directly influence ecosystem functioning under environmental changes, as previously reported for aboveground biodiversity^20^.

It has already been shown that climate disturbances have important impacts on N dynamics in ecosystems^21,22^ with potential legacy effects on ecosystem functioning and resilience to further disturbances^23–25^. In general, plant and microbial responses to climate disturbances vary between communities depending on the functional traits of the constituents^26,27^. Thus, environmental changes might alter soil biogeochemical functioning and N cycling through their effects on microbial abundance, diversity and community composition^21,22,28–30^.

An important step in the N cycle is the hydrolysis of organic molecules where N is released from its bound forms and made bioavailable^31^. Proteins, chitin and peptidoglycan are quantitatively the most important N containing molecules in soil^32^ and, therefore, their hydrolysis is important for N-related ecosystem functioning such as plant N provisioning. The use of degenerated oligonucleotides targeting the functional genes *alkaline (apr)* and *neutral (npr) metallopeptidases* as well as *serine peptidases* (*sub*)^33^, encoding for soil proteases, allows to trace the abundance, diversity and composition of the proteolytic microbial communities in soil^34,35^. In a previous soil incubation experiment, *apr* encoding microbial community composition showed different composition, higher diversity and enhanced stability under drought stress in organically versus conventionally managed soil^36^. No such effect was found for *npr* encoding microbial community diversity and composition^36^. In a parallel plant nutritional experiment, microbial communities in organically managed soil better provided N from a ^15^N labeled green manure to a standard phytometer compared to soil microbial communities in conventional managed soil under drought stress^36^. These results experimentally demonstrated that parts of the proteolytic microbial communities selected under organic farming can improve the capacity to maintain plant nutrition of a model crop under dry conditions^36^.

However, general knowledge about the effect of management on proteolytic microbial communities and associated ecosystem processes is still lacking. Thus, the present study aims to analyze and link N-related microbial communities with two important N-related agroecosystem processes (forage-N uptake and NO_3_^−^ leaching) in differently managed (conventional intensive *vs*. ecological intensive) agricultural systems under different simulated rain regimes. We conducted a terrestrial model ecosystem (TME) incubation experiment with soil and plants from paired, i.e. conventional intensively and ecological intensively managed, agroecosystems across Europe (mountain grassland in France, agroforest in Portugal and arable land in Switzerland). The TMEs were subjected to manipulated rainfall variability (normal, wet, dry and intermittent) in a laboratory experiment. N-related microbial communities were characterized by *apr* and *npr* abundance, *apr* diversity and composition as well as enzymatic activity involved in degradation of N containing molecules - leucine aminopeptidase activity (LAP) for protein degradation potential and β−1,4-N-acetylglucosaminidase (NAG) for chitin and peptidoglycan degradation^37^.

**Figure 1:**
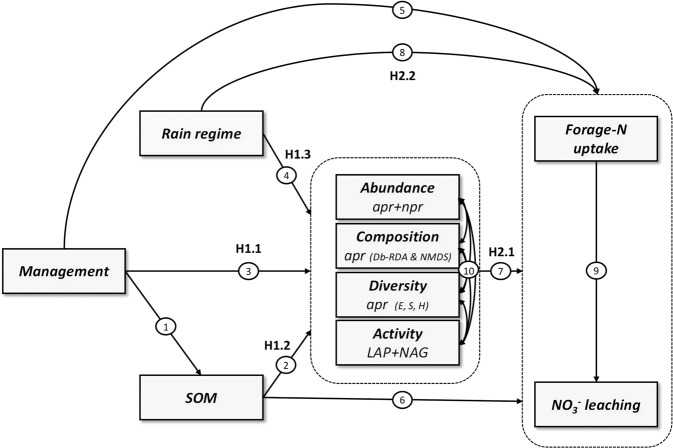
*A priori* models tested with structural equation modelling (SEM). Arrows ending/starting on/from the dotted box indicate paths ending/starting on/from all variables within the box. Our causal structure implies that management can affect the nitrogen (N)-related microbial community indirectly through modification of soil organic matter (SOM) concentration (arrows 1 and 2) or directly (e.g. plant traits or disturbance regime, arrow 3). By driving water availability, rain regime can directly influence microbial abundance/activity and community composition (arrow 4). N-related microbial communities can affect N-cycling processes (arrow 7) through the regulation of N released from organic matter. SOM concentration can influence forage-N uptake and NO_3_^−^ leaching through its effect on water and nutrient retention (arrow 6). A direct path between management and N-cycling processes was added to represent properties not included in our model (e.g. plant diversity or trait, arrow 5). Rain regime can directly affect forage-N uptake and NO_3_^−^ leaching by driving plant water availability and potentially exceeding soil retention capacity (arrow 8). Forage-N uptake can buffer NO_3_^−^ leaching by removing N from the soil (arrow 9). Free correlations between each pair of properties of N-related microbial communities have been added to represent potential covariation due to other causes than SOM concentration, management or rain regime (arrows 10). One-headed arrows represent causal relationships; double-headed arrows represent free correlations. Diversity indices: E = evenness, S = richness, H = Shannon diversity. Activity: LAP = leucine aminopeptidase extracellular enzyme activities, NAG = β-1,4-N-acetylglucosaminidase. Abundance: a*pr* = *alkaline metallopeptidase, npr* = *neutral metallopeptidase*. NMDS = non-metric multidimensional scaling, db-RDA = distance based redundancy analysis.

We hypothesize that rain regime and management affect forage-N uptake and NO_3_^−^ leaching across countries, directly and/or indirectly via modifications of N-related microbial communities (Fig. 1).

In detail we hypothesize:

I. Management system (ecological-intensive *vs*. conventional-intensive; H1.1), soil organic matter (SOM) (H1.2) and rain regime (wet, dry, intermittent *vs*. normal; H1.3) have direct effects on N-related microbial community abundance, activity, diversity and composition (Fig. 1).
  More precisely, ecological-intensive management, higher SOM concentration and wet rain regime are expected to positively affect N-related microbial community abundance, activity, diversity and community composition. Dry rain regime is expected to have an opposite effect with lower abundance, activity, diversity and shifted community composition whereas the intermittent rain regime might have no visible effect in regard to activity and abundance.
II. N-related ecosystem processes (i.e. forage-N uptake and NO_3_^−^ leaching) are influenced by management and SOM concentration mainly indirectly via the microbial community (Figs. 1, H2.1) and mainly directly by rain regime (Figs. 1, H2.2).
  More precisely, ecological-intensive management and high SOM concentration are expected to improve forage-N uptake via changes in the N-related microbial community (Figs. 1, H1.1 and H1.2). Highest forage-N uptake and NO_3_^−^ leaching is expected under wet, followed by intermittent and normal rain regime whereas lowest uptake and NO_3_^−^ leaching are expected under dry rain regime mainly due to direct effects from water scarcity independent of N-related microbial community.

## Materials and Methods

### Experimental set up and incubation conditions

Terrestrial model ecosystems (TMEs), which are defined as controlled, reproducible systems that attempt to simulate processes and interactions in a portion of the terrestrial ecosystem, were extracted from paired contrastingly (i.e. ecological intensive versus conventional intensive) managed systems at three different sites in autumn 2015 using special stainless-steel-extraction tube and a hydraulic excavator (SI Fig. 1). During the time of sampling the three sites were grassland cultivated for forage in the form of clover-grass representing arable cropping systems (Switzerland, Therwil), mountain grasslands (France, Vercors) and agroforestry systems (Portugal, Montemor-o-Novo). Detailed information and characterization of the different sites, contrasting management and applied practices can be found in Table 1. Thirty-two intact soil cores (30 cm depth x 16.5 cm diameter) encased in high-density polyethylene tubes were taken per country, extracted from four plots under conventional intensive farming and four plots under ecological intensive management. After extraction, TMEs were transported in a refrigerated truck to the Laboratory of Soil Ecology and Ecotoxicology of Coimbra University. Upon arrival, TMEs were acclimatized in special carts^38^ for 64 days prior to the start of the experiment. The carts allowed creating a temperature gradient between the lower and upper part of the soil core with temperatures ranging between 12 °C and 14 °C at the bottom of the TMEs. The distribution of the TMEs in the carts followed a randomized design. The carts were placed inside a climate chamber with controlled air humidity (≈60%) and temperature (20 °C ± 2 °C), and with a 16:8 h light:dark photoperiod during the entire experiment for all TMEs. Each TME was set up with a Decagon moisture sensor (LabFerrer, Spain); soil moisture was recorded three times a week in the upper 20 cm and adjusted with artificial rain water^39^ according to the respective rain regime. After acclimatization, differential rain regimes were installed for 263 days, which is the number of days needed for the intermittent rain regime to undergo a wet cycle, a dry cycle and coming back to the normal level. The rain regimes were as follows (SI Fig. 2): normal, dry, wet and intermittent according to each soil’s maximum water holding capacity (mWHC), which had been determined beforehand on intact soil cores. Prior to the start (T_0_) of the differential rain regimes, the vegetation in the TMEs was cut to 5 cm high and the soil surface of each TME was lined with a 2 cm layer of crop residues originating from the plots where the TMEs had been collected. After 263 days since the differential rain regimes has started, two soil cores of 98 cm^3^ (5 cm diameter and 5 cm height) were collected from each TME. Soil was sieved at 5 mm mesh and stored at 4 °C or −20 °C until being shipped under cooled conditions to different laboratories responsible for determining different parameters.

**Table 1:** Characterization of sites and their contrasting management. MAT = mean annual temperature, MAP = mean annual precipitation, N = nitrogen, SOM = soil organic matter. The Swiss site is based on a seven year crop rotation and terrestrial model ecosystems have been extracted in the second year of the grass clover period. The crop rotation is identical in the two managements and composed of the main crops: potato, winter wheat, soybean, maize, winter wheat and grass clover. All management practices indicated with an * are specific for the two year grass clover period and can vary depending on the crops of the seven year crop rotation. ^+^ Soil was not tilled during the grass clover period but identically tilled between the two managements in the other phases of the rotation; except for more frequent mechanical weeding in ecological intensive. ^**§**^ Values are corresponding to the mean and the standard error (se) of four TMEs destructively sampled before the beginning of the altered rain regime simulations (T_0_) for each management within each country.

Country (site coordinates)	Land use	Study design	MAT, MAP	Soil type	Texture^§^	SOM concentration^§^	Management	N Fertilizer (average N kg ha^-1^ year^-1)^	Weed control	Tillage	Forage use	Vegetation cover / plant richness ^§^	References
Switzerland 47°30′N 7°33′E	Grassland in rotation	Experimental plots (BIOORG and CONMIN)	9.7 °C, 791 mm	Haplic Luvisol	Silt: 81% ± 1 Sand: 5% ±0.4 Clay: 14% ± 1	4.11% ± 0.33	Ecological intensive (since 37 years)	Slurry* (120)	Mechanical	^+^	Grass cut 4 times a year for livestock *	Grass: 39% ± 3 Legumes: 61% ± 3 Other: 0 Richness: 6 ± 0	^40^
					Silt: 83% ± 2 Sand: 4% ±0.3 Clay: 13% ± 2	4.19% ± 0.39	Conventional intensive (since 37 years)	Synthetic* (140)	Mechanical*	^+^	Grass cut 4 times a year for livestock *	Grass: 48% ± 4 Legumes: 51% ± 4 Other: 0 Richness: 6 ± 0	
France 45°07′N 5°31′E	Mountain grassland	Farm comparison	7.2 °C, 1483 mm	Orthic Luvisols	Silt: 35% ± 5 Sand: 49% ±7 Clay: 16% ± 2	9.01% ± 1.32	Ecological intensive (since 50 years)	Cow manure (30)	Absent	Absent	Grazing 1–3 times a year	Grass: 51% ± 16 Legumes: 12% ± 3 Other: 37% ± 16 Richness: 7.5 ± 0.7	^41^
					Silt: 41% ± 4 Sand: 47% ±5 Clay: 12% ± 1	9.54%± 1.25	Conventional intensive (since 50 years)	Cow manure (70)	Absent	Every 3–4 year	Grazing 0–1 times a year and mowed 1–2 times a year	Grass: 59% ± 13 Legumes: 36% ± 14 Other: 5 ± 2 Richness: 6 ± 2	
Portugal 38°42′N 8°19′W	Grassland in agroforest	Farm comparison	16.5 °C, 1093 mm	Histic- mesic Inceptisol	Silt: 21% ± 4 Sand: 70% ±4 Clay: 10% ± 1	3.45% ± 0.27	Ecological intensive (since 18 years)	None(0)	Mechanical	Absent	Planned grazing by cattle and pigs	Grass: 28% ± 7 Legumes: 5% ± 1 Other: 66% ± 7 Richness: 33 ± 0.5	^10^
					Silt: 23% ± 4 Sand: 65% ±5 Clay: 12% ± 1	3.65% ± 0.40	Conventional intensive (since 18 years)	Synthetic (56)	Mechanical	Every 2^nd^ year	Intensive grazing by sheep	Grass: 40 ± 2 Legumes: 10 ± 3 Other: 48 ± 5 Richness: 33 ± 2

**Figure 2:**
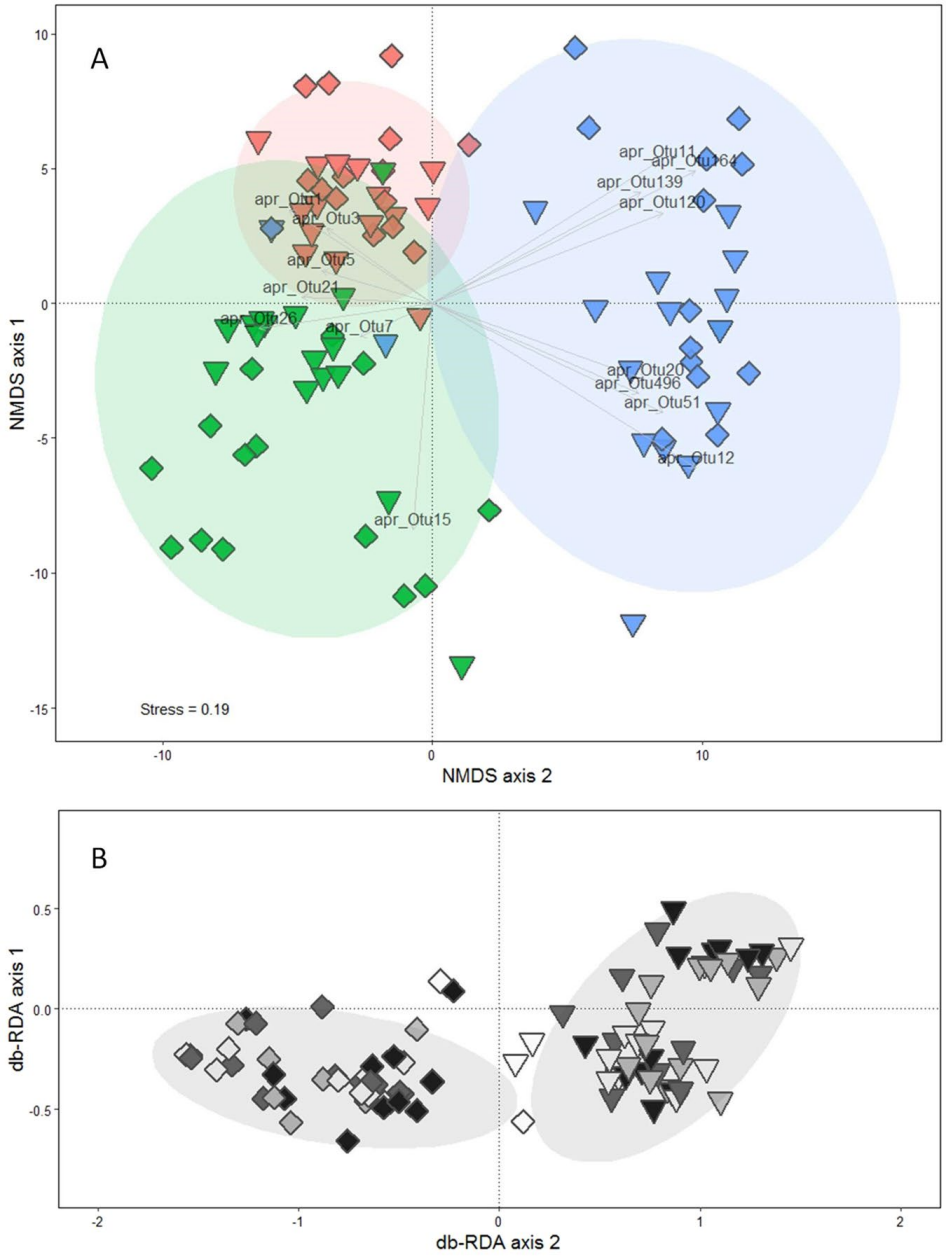
Overall proteolytic microbial community composition (**A**) and proteolytic microbial community composition under the influence of management only (**B**). Dissimilarity between *alkaline metallopeptidase* (*apr)* operational taxonomic units (OTUs) (97% sequence similarity) based on Bray-Curtis distance metrics are ordinated by nonmetric multidimensional scaling (NMDS) (A) and distance-based redundancy analysis (db-RDA) using the capscale function constraining for management and conditioning for country (**B**). Triangles represent ecological intensive management, and squares represent conventional intensive management. In A, the different symbol fills represent the different countries: red = Switzerland, green = France and blue = Portugal. In B, the different symbol fills represent the four rain regimes: black = dry, dark-grey = normal, light-grey = intermittent and white = flood. Ellipses represent the 95% confidence intervals of countries (**A**) and management (**B**), respectively. Vectors indicate OTUs being statistically influential for the differentiation between countries (identified *via* simper.pretty analysis and Kruskal tests with fdr p-value corrections).

Throughout the entire experiment forage-N uptake of each TME was monitored via cutting the vegetation of all TMEs down to 5 cm whenever the height in one treatment reached 20 cm in order to simulate grazing/cutting (13 harvests in total). Fresh plant material from each cut and each TME was weighted and dried at 40 °C for four days to assess aboveground dry weight. Soil leachates of each TME were collected periodically throughout the experiment (at one to two weeks intervals). After each collection, volumes were measured and leachates acidified before storage at −20 °C until being processed for nutrient analysis.

### Analysis of aboveground plant biomass

All individual aboveground vegetation cuts of a TME during the altered rain regime period were pooled and homogenized. Carbon (C) and N concentrations of dried (40 °C) and ball milled samples were then assessed by combustion (CN Vario Max; Elementar Analysen Systeme GmbH, Hanau, Germany). Forage-N uptake was calculated in kg N per day and hectare (ha) (1):

1 $$Forage-N\,uptake(kg\,N\,da{y}^{-1}h{a}^{-1})=\frac{Plant\,biomass(kg)\times plant\,N\,concentration( \% )}{Time(days)\times TME\,surface(ha)}$$

### Biogeochemical analyses of soil, leachates and plant litter

Soil organic matter (SOM) concentration was measured on a subsample of dry soil as loss on ignition at 550 °C for 4 h and expressed as percentage. Soil C and N content was measured using an elemental analyzer (FlashEA 1112, Fisher Scientific, Waltham, Massachusetts, USA) on oven-dried subsamples ground to a fine powder (5 µm diameter) with a ball mill (MM301, Retsch GmbH, Haan, Germany). Soil pH was determined in a 1:6 1 M KCl solution. Soil dissolved mineral and organic N were extracted from 3 g of dried soil using a 1:10 soil to CaCl_2_ (0.01 M) extraction followed by centrifugation and filtering through 0.45 μm cellulose acetate membrane filter^42^. Soil dissolved mineral N pools (NH_4_^+^, NO_3_^−^, NO_2_) were measured by automated colorimetry (Brann enLuebbeTrAAcs 800 Autoanalyzer; Skalar Analytical B.V., Breda, the Netherlands) and dissolved organic N (DON) and dissolved organic C (DOC) by a TOC-TN analyzer (Skalar Analytical B.V., Breda, the Netherlands). PO_4_^−^ was extracted from 10 g fresh soil using 0.5 M K_2_SO_4_ and measured on an automated photometric analyser using standard colorimetric methods (Gallery Plus: Thermo Fisher Scientific, Waltham, Massachusetts, USA) and total soil P (P) was measured using Mehlich method. NO_3_^−^ content in leachates was measured on an automated photometric analyzer (Gallery Plus: Thermo Fisher Scientific, Waltham, Massachusetts, USA). NO_3_^−^ leaching was assessed by calculating the total amount of NO_3_^−^ lost from each TME per day and hectare (2).

2 $$N{O}_{3}^{-}\,leaching(mg\,N{O}_{3}^{-}-N\,da{y}^{-1}h{a}^{-1})=\frac{Volume(L)\times N{O}_{3}^{-}\,concentration(mg\,N{O}_{3}^{-}-N\,{L}^{-1})}{Time(days)\times TME\,surface(ha)}$$

Naturally senesced leaf litter of the plant species was collected at the end of summer 2015 in the respective sites. The leaf litter was air-dried for one month, grounded and sent for chemical analyses. Leaf litter N and C concentrations were analyzed using an elemental analyzer (Flash 1112 EA, Thermo-153 Finnigan, Bremen, Germany), while leaf litter P concentration was analyzed by atomic absorption spectrometry (Perkin Elmer ICP-OES 6500, Norwalk, USA). Leaf litter cellulose and lignin were assessed by the method of Van Soest^43^. Several stoichiometric indices were calculated: C:N, lignin:N and lignin:P ratios, as well as the lignocellulose index (LCI = lignocellulose index = lignin/[lignin + cellulose]).

## Microbial analyses

### Enzyme activity

Leucine aminopeptidase activity (LAP) and β−1,4-N-acetylglucosaminidase (NAG) potential activities were estimated using standard fluorimetric techniques^44^. Briefly, 2.75 g of soil was homogenized (1 min in a Waring blender) in 200 ml of a sodium acetate buffer solution adjusted to the mean pH (5.1 ± 0.7 SD, n=24) of soil samples measured at T_0_. The soil slurry (800 µL) was then added in technical duplicates to the appropriate wells of a 96-deep-well microplate with 200 µL of substrate specific to the two targeted enzymes at saturation concentration. For each soil sample, duplicated standard curves (0-100 µM concentration) were prepared by mixing 800 µL of soil slurry with 200 µL of 4-methylumbelliferone (MUB) or 7-amino-4-methylcoumarin (MUC) in 96-deep-well microplates. Plates were incubated at 20 °C in the dark (3 h) on a rotary shaker (150 rpm) before centrifugation at 2900 g (3 min). The supernatant (250 µL) was transferred to a black Greiner flat-bottomed plate and fluorescence was measured on a microplate reader (Varioscan Flash, Thermo Scientific) with excitation wavelength set to 365 nm and emission set to 450 nm. After correcting for negative controls, potential enzyme activities were expressed as nmol g soil^−1^ h^−1^. Activities of NAG and LAP were summed to represent the total potential activity of N-rich molecule hydrolysis (proteins, chitin and peptidoglycan).

### Abundance of proteolytic microbial communities

Abundance of alkaline (*apr*) and neutral (*npr*) metallopeptidase genes was assessed by quantitative polymerase chain reaction (qPCR) using degenerated oligonucleotides^33^. Beforehand, DNA was isolated from lyophilized soil samples using the FastDNA SPIN Kit for Soil (96×) (MP Biomedicals, CA, USA). Prior to DNA extraction each sample was spiked with an exact amount of plasmid carrying an artificial sequence to normalize DNA extraction efficiency rates between the samples and to test for presence of PCR inhibitors. DNA concentrations were measured using a Qubit Fluorometer (Thermo Fisher Scientific, Waltham, USA). Prior to qPCR, cycling conditions of oligonucleotides were optimized using different soil DNA dilutions and annealing temperatures to reach standard curves with an R^2^ > 0.999 and amplification efficiencies between 0.8 and 1 (SI Table 1). qPCR reactions were performed using a SYBR green approach (Kapa SYBR Fast qPCR Kit Master Mix (2×) Universal; Kapa Biosystems, Wilmington, MA, USA) on a CFX96 Touch Real-Time PCR Detection System (Bio-Rad, Switzerland). Biological replicates were analyzed in technical duplicates. Negative controls and serial dilutions of plasmids carrying the gene of interest were included as triplicates to calculate standard curves for absolute quantification. Size and quality of generated amplicons were controlled by melting curve analyses and gel electrophoresis. Due to the low abundance of *npr* genes, community diversity and composition was exclusively assessed for *apr* and not *npr* encoding microbial communities.

### Composition and diversity of proteolytic microbial communities

DNA extracts were processed in a two-step PCR approach using fluidigm tagged *apr* primers for the first PCR performed in quadruplicates. Oligonucleotide sequences and cycling conditions are listed in SI Table 1. PCR quadruplicates of each sample were subsequently pooled and loaded on agarose gels (1.75%) for visualization and validation. Target bands were cut out and purified using the QIAquick gel extraction kit (QIAGEN, Switzerland). The subsequent second PCR, library preparation and sequencing on an Illumina MiSeq sequencing system using the 2×250 bp Reagent Kit v2 (Illumina, San Diego, CA, USA), was performed at the Genome Quebec Innovation Center (Montreal, Canada) according to the amplicon guidelines provided by Illumina. The FastQC^45^ and MultiQC^46^ tools were used to assess quality of the sequencing data. Forward and reverse sequences were merged (overlap up to 250 base pairs) using Flash^47^. Operational taxonomic unit (OTU) clustering on 97% similarity was performed using UPARSE^48^ and taxonomic annotation of OTUs was performed within MGX^49^. The raw sequences are openly deposited at the European Nucleotide Archive under the accession number PRJEB33546.

### Statistical analyses

Hereafter, forage-N uptake and NO_3_^−^ leaching are defined as “N-cycling processes” while NH_4_^+^, NO_3_^−^ + NO_2_, and DON content are defined as “N-related soil indicators”. The term “N-related microbial indicators” is composed of the abundance, diversity and composition of proteolytic microbial communities as well as the extracellular enzymatic activity potential degrading organic N rich substrates (NAG + LAP). Treatment effects on N-cycling processes as well as N-related soil and microbial indicators were first assessed using linear mixed effect models and then integrated and linked in a hypothetical causal network using structural equation modeling (SEM).

### Proteolytic microbial community analyses

In order to assess variations in proteolytic microbial community composition, *apr* OTU abundances were subjected to a Hellinger transformation using the ‘decostand’ function implemented in the vegan package^50^. Overall variation in proteolytic microbial community composition was visualized using nonmetric multidimensional scaling (NMDS) based on Bray-Curtis distance metrics. Permutational multivariate analysis of variance (PERMANOVA) was then performed using the ‘adonis’ function with country used as strata to statistically assess treatment effects on the distance matrix within each country. Significant factors of the PERMANOVA were implemented as constraining terms in a distance-based redundancy analysis (db-RDA) using the capscale function^50^ with Bray-Curtis distance metrics and country set as a conditioning term. This db-RDA was used to identify the sub-part of microbial community composition under the influence of treatments. Analysis of variance (ANOVA) was used to test for treatment effects of the capscale object in the db-RDA. Both ordinations were computed using the vegan package^50^.

OTUs being most important in differentiating between treatments were assessed with the ‘simper.pretty’ and ‘kruskal.pretty’ function^51^ using false discovery rate corrections. Only OTUs significantly differing between treatments were selected for biplotting in the ordinations. All analyses were run on R 3.4.1^52^.

Alpha-diversity was assessed based on richness, evenness and Shannon index calculated using UPARSE^48^. Coordinates of the ordinations (NMDS and db-RDA) were extracted and used in the following statistical analyses as proxy of the proteolytic microbial community composition. Coordinates extracted from the NMDS were used as indicator of the overall variation while coordinates from db-RDA were used as indicators of the sub-part of microbial community composition under the influence of treatments (constrained composition).

### Rain regime and management effects (mixed effects models)

Effects of rain regime and management on N-cycling processes and on N-related soil and microbial indicators were assessed using mixed effect models with rain regime and management as fixed factors, country and plot as random factors. Plot was nested in management and together they were nested in country to take into account the nested design of the experiment^53^. Data were transformed using log, square-root or inverse functions to satisfy the assumption of normal distribution and variance homogeneity of model residuals when necessary. Post-hoc comparisons were done using Tukey’s honest significant difference test. Mixed effect models and post-hoc comparisons were run under R.3.5.1^52^ using the nlme^54^ and lsmeans^55^ packages.

### Direct and indirect effects of management and rain regime on N-cycling processes (SEM)

Piecewise SEM model selection^56–58^ was used to identify the best causal network explaining rain regime, management and SOM effects on N-cycling processes through N-related microbial communities. Exploratory SEM is useful when systems have been poorly studied yet and can help to identify main mechanisms within a series of potential mechanisms hypothesized based on current knowledge^56,57^. Shipley’s test of d-separation^59^ was used to assess if missing paths in the hypothesized structure exist. Next, a d-separation test was used to generate Fisher’s C statistic for the overall SEM^58,59^. Herewith identified significant p-values indicate that the hypothesized structure is wrong - in other words: some other paths not included in the hypothesized structure exist. Piecewise SEM makes results less sensitive to sample size and enables to include mixed effect models within the SEM structure^58^. Based on results from a previous experiment with the Swiss study site, supporting the control of N-cycling processes by the N-related microbial community^36^, we built a hypothesized causal model with microbial communities affecting forage-N uptake and NO_3_^−^ leaching and not *vice versa* (forage-N uptake and NO_3_^−^ leaching controlling microbial community). We used a model selection process to obtain the most parsimonious model depicting rain regime and management effects on N-cycling processes through the N-related microbial community. Initially, we fitted the full model containing all potential paths of our *a priori* model (Fig. 1). Next, a first simplification process was conducted by removing variables without any significant relationship to rain regime, management, SOM or N-cycling processes (non-informative variables). Then, we simplified this model by removing relationships between remaining variables starting with less significant relationships so as to retain the most significant ones. Each path removal was accepted if the model quality based information-theoretic criterion (BIC) was improved^60^. After the most parsimonious model was obtained, global model fit and quality were verified using Fisher’s C test and R² of endogenous variables before interpreting path coefficients as suggested by Hertzog^60^. Lastly, we tested and present a second model, focusing on N-cycling drivers, by removing all variables without any significant relationship with N-cycling processes. All variables were transformed with log, square root or inverse log functions to respect normality of residuals. Furthermore, all paths were standardized. All models in the SEM used plot nested in management and together they were nested in country. Analyses were conducted using piecewiseSEM package^58^ run under R.3.5.1^52^.

### Identification of variables potentially driving the management effect on proteolytic (*apr*) microbial community composition

Additional analyses were conducted to assess a potential relationship between soil properties, litter properties, vegetation composition and vegetation diversity with the sub-part of the proteolytic microbial community composition under the influence of management (coordinates of the first axis of the db-RDA measured in TMEs subjected to normal rain regime). Vegetation composition and vegetation diversity as well as litter traits have been measured *in situ* at the plot level^10^, from where the TMEs of the current experiment were obtained; soil properties were measured in the TMEs at the start of the experiment (texture) or in the same soil core as soil microbial community composition. Linear regression between the respective variables and proteolytic (*apr)* microbial community composition was conducted using country as random factor. All variables were also tested for potential management effects using ANOVA such as mixed effect models using country as random factor.

## Results

### Rain regime and management effects on N-cycling processes and N-related soil indicators

Forage-N uptake increased from dry to normal and from normal to wet treatments while the intermittent treatment did not significantly differ from dry and normal treatments (SI Fig. 3). NO_3_^−^ leaching increased from dry to normal treatments and from normal to intermittent and wet treatments (SI Fig. 3). Soil NH_4_^+^ and NO_3_^−^ + NO_2_^−^ concentrations were significantly affected by rain regime, and DON content marginally so (Table 2). Soil NH_4_^+^ increased under dry treatments while NO_3_^−^ + NO_2_^−^ content decreased under wet compared to normal treatments (SI Fig. 3).

**Figure 3:**
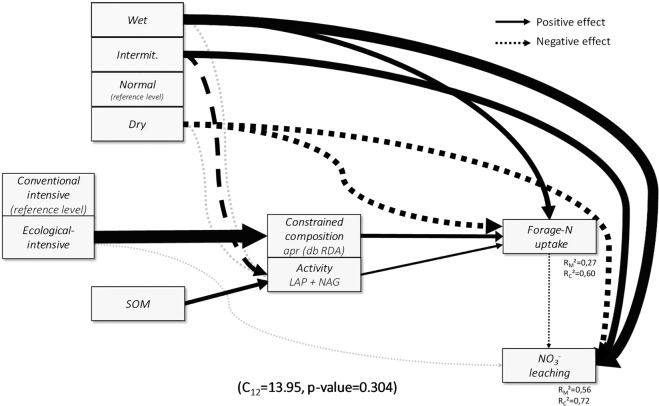
Structural equation model (SEM) representing paths from rain regime and management to nitrogen (N)-cycling processes through soil organic matter (SOM) concentration and N-related microbial communities. Arrow width represents standardized effect size, black arrows represent significant paths, light grey arrows represent non-significant paths conserved during model selection process (see SI Table 3 for all coefficient values and significance, and Figure SI 4 for the full model including also N-related microbial properties not affecting N-cycling processes). Marginal R² (R²_m_) and conditional R² (R²_c_) are given only for ecosystem processes (see SI Table 3 for R² of all endogenous variables). One-headed arrows represent causal relationships. LAP = leucine aminopeptidase, NAG = β-1,4-N-acetylglucosaminidase, *apr* = *alkaline metallopeptidase*, db-RDA = distance based redundancy analysis. ‘Constrained composition *apr* (db-RDA)’= projected score of the first db-RDA axis of proteolytic *(apr)* microbial community composition (representing the sub part of the composition constrained by management).

**Table 2:** Effects of rain regime (RR) and management (M) on nitrogen (N)-cycling processes and N-related soil and microbial indicators. Effects were assessed by a mixed effects model using rain regime and management as fixed effects and plot nested in country as random factor. R²m = marginal R² representing the variation explained by fixed factors (RR and M), R² = conditional R² representing the variation explained by fixed (RR and M) and random factors (Country and Plot). N = nitrogen, SOM = soil organic matter, DON = dissolved organic nitrogen, LAP = leucine aminopeptidase extracellular enzyme activities, NAG = β-1,4-N-acetylglucosaminidase extracellular enzyme activities, *apr = alkaline metallopeptidase*, *npr= neutral metallopeptidase*. Df = degrees of freedom.

Parameter	Rain regime		Management		RR X M		R²_m_	R²_c_
	Df (3,66)		Df (1,2)		Df (3,66)			
	F	p-value	F	p-value	F	p-value		
***N-cycling processes***								
Forage N uptake	11.01	<0.001	0.21	0.691	0.53	0.665	0.22	0.40
NO_3_^−^ leaching	53.88	<0.002	1.55	0.340	1.55	0.210	0.59	0.67
***N-related soil indicators***								
SOM	7.06	<0.001	0.16	0.730	0.51	0.680	0.02	0.94
Total Soil N	1.39	0.255	1.19	0.389	0.30	0.828	0.03	0.79
NH_4_^+^	15.08	<0.001	0.49	0.556	1.45	0.235	0.14	0.76
NO_3_^−^ + NO_2_^−^	3.81	0.014	0.68	0.495	0.57	0.638	0.09	0.39
DON	2.62	0.058	0.84	0.456	1.61	0.196	0.11	0.33
***N-related microbial indicators***								
Activity (LAP + NAG)	2.90	0.041	0.67	0.499	0.61	0.613	0.07	0.43
Abundance (*apr* + *npr*)	0.63	0.600	0.03	0.870	2.08	0.110	0.03	0.70
Richness (*apr*)	1.73	0.170	0.01	0.932	0.85	0.472	0.05	0.39
Shannon diversity (*apr*)	1.37	0.259	0.18	0.712	0.46	0.709	0.05	0.28

### Rain regime and management effects on N-related microbial indicators

Enzymatic activity (LAP + NAG) was affected by rain regime (Table 2, Fig. 3). Highest values were found in normal (371.7 nmol g soil^−1^ h^−1^), followed by low (338.9 nmol g soil^−1^ h^−1^) and wet rain regimes (300.0 nmol g soil^−1^ h^−1^) while lowest values were identified in intermittent rain regime (251.0 nmol g soil^−1^ h^−1^). Abundance of *apr* + *npr* as well as *apr* richness and Shannon diversity were neither affected by management nor by rain regime (Table 2).

Sequencing of proteolytic (*apr*) microbial communities revealed in total 25023 different OTUs with 97% sequence similarity (OTU list can be found in SI File 1). Overall assessment of proteolytic (*apr*) microbial community composition using NMDS showed strong variation across countries (Fig. 2A). PERMANOVA showed management effects but no rain regime effect (Table 3, country specific results can be found in SI Table 2). Next, a db-RDA ordination conditioning for countries was used to depict proteolytic (*apr*) microbial community composition only under the influence of management (Fig. 2B). ANOVA on the capscale object of the db-RDA confirmed the significant management effect (Table 3, p= 0.002). Using the simper.pretty function, a total of 18 single *apr* OTUs being most important in discriminating between countries could be identified (Fig. 2A) but could not get assigned to sequences yet known/described. Since significant *apr* OTUs were found specifically for each country it is assumed that no bias, such as chimeric products, was present but rather that databases are still not complete/deep enough to assign functional gene sequences to taxa. OTUs significantly discriminating between management or rain regime were not found. Overall, only approximately one third of all *apr* OTUs could be assigned to already identified sequences, which were dominated by *Pseudomonas* (SI File 1), while two thirds are of yet unknown sources.

**Table 3:** Overall effects of rain regime and management on proteolytic microbial community composition. Effects were assessed by PERMANOVA (999 permutations) on a distance matrix based on *alkaline metallopeptidase* (*apr*) operational taxonomic units (OTUs) using Bray-Curtis distance metrics and with country as strata to assess treatment effect across countries. Df = degree of freedom, n.s = non-significant.

	Df	F	R^2^	p
Rain regime	3	0.98	0.03	n.s
**Management**	1	1.34	0.01	**0.001**
Management x Rain regime	3	0.95	0.03	n.s

### Direct and indirect effects of management, SOM and rain regime on N-cycling processes *via* soil microbial community modifications

The SEM selection process led to the removal of *apr* evenness and Shannon diversity indices from the final model since neither were significantly related to rain regime, management, SOM or N-cycling processes. Next, model simplification using BIC criterion enabled a large model improvement (Initial BIC = 364.49, Final BIC = 302.52). Simplified SEM structure showed good fit with observations (indicated by Fisher’s C test p-value higher than 0.05: C_48_ = 53.22, p-value= 0.28, SI Figure 4). The final SEM includes fourteen significant paths. Endogenous variables (overall proteolytic (*apr*) microbial composition, constrained proteolytic (*apr*) microbial composition, richness (*apr*), activity (LAP + NAG), abundance (*apr* + *npr*), forage-N uptake, NO_3_^−^ leaching) showed marginal R² (fixed factors effect) ranging from 0.09 to 0.80 (mean= 0.39) and conditional R² (fixed and random factors effects) ranging from 0.32 to 0.88 (mean = 0.61) (see SI Table 3 for details). Finally, after removing all variables without any significant relationship with N-cycling processes, the final model focusing on N-cycling process drivers still showed a good fit (C_12_ = 13.95, p-value = 0.304, Fig. 3). SEM analysis confirmed that management, rain regime and SOM concentration influenced N-cycling processes. Rain regime did directly and indirectly affect N-cycling processes whereas exclusively indirect effects were observed for SOM and management on N-cycling processes through modifications of N-related microbial communities. Conditional R²s were 0.60 and 0.72 for forage-N uptake and NO_3_^−^ leaching respectively, and indicated that more than half of the variation in N-cycling processes was explained by our models. Marginal R², representing the variation explained by fixed factors only (rain regimes, management, soil and microbial properties), were 0.27 and 0.56 for forage N-uptake and NO_3_^−^ leaching, respectively.

Altogether, SEM depicted four major paths (Fig. 3, SI Figure 4). Firstly, SEM analysis indicated strong direct effects of rain regime on N-cycling processes. Wet and intermittent rain regime increased NO_3_^−^ leaching compared to normal rain regime (used as reference) whereas drought decreased NO_3_^−^ leaching. Furthermore, wet directly increased and dry decreased forage-N uptake compared to normal rain regime. Secondly, SEM analyses indicated indirect effects of rain regime on N-cycling processes via modification of enzymatic activity. Intermittent rain regime decreased forage-N uptake via negative effects on microbial activity (LAP + NAG). Thirdly, SEM indicated an indirect positive effect of ecological intensive management (compared to conventional intensive management as reference) on N-cycling processes through an effect on proteolytic (*apr*) microbial community composition represented by the db-RDA coordinates. Microbial community composition in ecological intensively managed soils had a positive effect on forage-N uptake, which in turn translated into decreased NO_3_^−^ leaching. Lastly, SEM showed that SOM concentration was a main driver in shaping N-related microbial communities *via* an increase in the activity (LAP + NAG) (Fig. 3, SI Figure 4), abundance (*apr* + *npr*) and richness (*apr*) as well as a modification of the overall proteolytic (*apr*) microbial community composition (represented by NMDS coordinates) (SI Figure 4). Furthermore, a positive indirect effect of SOM concentration on forage-N uptake *via* microbial activity (LAP + NAG) was observed (Fig. 3, SI Figure 4).

### Identification of variables potentially involved in driving the management effect on proteolytic microbial community composition

Management significantly affected P-associated litter traits, with higher litter-P content and lower litter C:P, N:P as well as lignin:P ratios in ecological intensively compared to conventional intensively managed systems (Table 4) whereas no such effects on soil properties were found (SI Table 4). Management effects on the vegetation composition in ecological intensively managed systems were found only for plant cover in the group “other” (non-grass and non-leguminous species) (Table 4). High conditional R² and low marginal R² observed for litter-P content, litter C:P and lignin:P ratio as well as the amount of non-grass and non-leguminous species indicated a large influence of country compared to management. Conversely, litter N:P ratio variation was mostly explained by management and only marginally by country.

**Table 4:** Management effect on litter and vegetation properties and their correlation with proteolytic (*apr*) microbial community composition assessed using mixed effect model with country as random factor. R²_m_ = marginal R², R²_c_ = conditional R²_c._ ADF = acid detergent fibre, ADL = acid detergent lignin, LCI = lignocellulose index (lignin/(lignin + cellulose), C = carbon, N = nitrogen, P = phosphorus, db-RDA = distance based redundancy analyses, *apr*= *alkaline metallopeptidase*.

Plant community properties	Management effect			Correlation with constrained *apr* composition (db-RDA axis 1)		
	p	R²_m_	R²_c_	p	R²_m_	R²_c_
***Litter Trait***						
ADF (% dry mass)	0.0703	0.01	0.97	0.552	0.02	0.02
ADL (% dry mass)	0.4786	0.00	0.99	0.652	0.01	0.01
Cellulose (% dry mass)	0.3157	0.01	0.73	0.793	0.00	0.00
LCI	0.7709	0.00	0.94	0.744	0.00	0.00
C (% dry mass)	0.7193	0.00	0.97	0.623	0.01	0.01
N (% dry mass)	0.2209	0.00	0.97	0.551	0.02	0.02
P (% dry mass)	0.0362	0.05	0.79	0.007	0.31	0.72
C:N	0.8610	0.00	0.95	0.544	0.02	0.02
N:P	0.0017	0.32	0.43	0.003	0.32	0.32
C:P	0.0001	0.06	0.95	0.006	0.33	0.87
lignin:N	0.0917	0.00	0.96	0.863	0.00	0.00
lignin:P	0.0007	0.06	0.92	0.313	0.04	0.04
***Vegetation composition and diversity***						
Legumes cover (%)	0.2320	0.02	0.74	0.768	0.00	0.00
Grass cover (%)	0.1806	0.06	0.24	0.168	0.08	0.08
Others (non-grass, non-legume) cover (%)	0.0179	0.07	0.77	0.009	0.29	0.68
Plant richness	0.3403	0.00	0.98	0.757	0.00	0.00
Plant Shannon diversity	0.3292	0.01	0.82	0.873	0.00	0.00

Except for lignin:P ratio, all litter and vegetation parameters affected by management were significantly correlated with the sub-part of proteolytic (*apr)* microbial community composition under the influence of management (constrained composition, db-RDA axis 1) (Table 4). Litter-P content, litter C:P and litter N:P ratio as well as the amount of non-grass and non-leguminous species explained almost equivalent parts of the proteolytic (*apr*) microbial community composition with marginal R²s around 0.30. Contrary, the correlation between proteolytic (*apr*) microbial community composition and litter N:P ratio presented no country effects (marginal R² equal to conditional R²), indicating that the intercept of the correlation between litter N:P ratio and *apr* composition was not conditioned by country. Thus, litter N:P ratio was more consistent between countries than the other plant properties and a better candidate to be a possible driver of proteolytic (*apr*) microbial community composition.

## Discussion

Management, SOM, rainfall variability and climate change affect soil microbial communities, biogeochemical cycling and thus soil fertility and ecosystem services in agro-ecosystems^61,62^. The current study aimed at assessing the effects of contrasting rain regimes (dry, wet, intermittent *vs*. normal), management (ecological intensive *vs*. conventional intensive) and SOM concentration on N-related ecosystem processes partially mediated by microbes, in forage agroecosystems across three European countries.

In general, results of our TME incubation study confirmed the overall hypotheses (Fig. 1) stating that across countries, rain regime, management and SOM directly and/or indirectly *via* modifications of N-related microbial communities, affect forage-N uptake and NO_3_^−^ leaching (Fig. 3). Ecological intensive management influenced exclusively N-related microbial community composition and not abundance, activity and diversity (Tables 2 and 3) and thus hypothesis H1.1 was validated for composition but not for other microbial community parameters. SOM positively affected N-related microbial community abundance, activity and diversity and modified community composition (Fig. 3, SI Figure 4), which is in agreement with hypothesis H1.2. Rain regime exclusively affected N-related microbial enzymatic activities but not abundance, diversity and composition (Tables 2 and 3) indicating high resistance of N-related microbial community, and these results being partially in agreement with hypothesis H1.3 (Fig. 1). Finally, our results are in agreement with our second hypothesis (Fig. 1) that N-related ecosystem processes are positively influenced mainly directly by rain regime (H2.2), and by ecological-intensive management and SOM mainly indirectly *via* N-related microbial communities (H2.1). Overall our results provide new insights into potential mechanisms controlling agro-ecosystem functioning under projected climate changes. Hereafter, we first discuss how management and SOM indirectly influence N-related ecosystem processes through modification of proteolytic (*apr)* microbial community composition. Subsequently, we discuss how rain regime directly affects N-related ecosystem processes and soil indicators, but only to a lesser extent microbial communities.

### Management and SOM influence N-related ecosystem processes through modification of N-related microbial communities

During the last twenty years many studies assessed long-term management effects on the microbial community composition in soil^12,63^. Yet, only few studies focused on a functional group of microorganisms and even less have assessed potential repercussions on ecosystem processes. In the present study found effects of management on proteolytic (*apr*) microbial community composition (Fig. 2, Table 3) and SEM depicted ecological intensive management to affect proteolytic microbial community composition promoting forage-N uptake and buffering NO_3_^−^ leaching (Fig. 3). These results support the findings from a previous laboratory study on soils of the Swiss site^34^ with extended experimental systems closer to ecological realism, across a wider range of management and pedo-climatic conditions. However, even though good experimental support^36^ for our SEM causal structure exists (Fig. 1), results should be considered as a potential causal model rather than a proof of causality^57^. Furthermore, the proteolytic microbial community might *vice versa* has also been affected by N-related processes as plants can affect the associated root microbiome^64–66^. Our experimental set up did not allow to disentangle which effect was present but as microbial communities in bulk soil and not rhizosphere were studied, and because we have theoretical^67^ and previous experimental evidences^36^, we interpret our results as one-directional, even though cautiously.

The fact that management effects on N-cycling processes occur through *apr* composition, and not *via* other proteolytic microbial community properties such as *apr* abundance or diversity stresses that not only “how many functional genes” (abundance of a functional group) or “how many functional OTUs” (diversity within a functional group) matters but also “which functional OTUs” (composition of the functional group)^61^. The involvement of proteolytic microbial community composition in promoting N-related ecosystem processes might be explained by distinct traits associated to certain organisms/species carrying the *apr* sequence – some organisms might encode for more efficiently or differently working proteases, produce other enzymes involved in N-hydrolysis or even harbor other traits favoring forage-N uptake. Furthermore, the presence of *apr* sequences within organisms is only a discrete trait indicating a potential to produce the respective enzyme. Thus, moving forward to realized and continuous trait measurement^68^ by relating taxa to enzyme production and kinetics could help to elucidate why and when functional gene encoding microbial community composition matters. Additionally, functional screening using isolation and physiological characterization^69^ as well as shotgun metagenomics and metatranscriptomics assessing the entire metabolic potential of a given community^70^ could help to bridge parts of this gap. When analyzing proteolytic microbial community composition in detail, we found *apr* OTUs discriminating between countries, whereas across all countries no *apr* OTUs significantly discriminating between managements could be found. Overall, only one third of *apr* OTUs could be assigned to known sequences/database entries, calling for future research to identify hidden functional players in the proteolytic gene pool. The annotated sequences were dominated by *Pseudomonas*, which is in line with the current literature^36,71^. However, annotation also revealed sequences to be associated to organisms outside the prokaryotes. In general, results of amplicon sequencing targeting functional genes should be interpreted cautiously due to some limitations^72^. Even though highly degenerated, primers might miss certain sequences and thus diversity and composition might be under- or overestimated. Furthermore, PCR, library preparation, sequencing, and especially annotation bear further bias. Besides *apr*, also *npr* targets protease encoding microbial communities are functionally involved in N-related ecosystem processes but have been shown to be less responsive to water treatments^36^. Additionally, *serine peptidases* play an important role in organic-N hydrolysis^33,73^ but were not investigated in this experiment. By only looking at a selected part of the proteolytic microbial community, we probably missed important effects. However, even though exclusively looking at a subset of the proteolytic microbial community, our results suggest a central role of proteolytic microbial communities in regulating the N cycling in the context of climate change. Thus we call for a deeper characterization of this functional group by also analyzing *npr* and *sub* encoding proteolytic microbial communities as well as by moving forward and investigating their realized traits to understand why and where proteolytic community composition matter for ecosystem functioning.

In the present study no management effect on SOM concentration was found (Table 2) indicating management to affect proteolytic microbial community composition independent of SOM concentration. The absence of management effects on SOM in the current study is inconsistent with recent global scale meta-analyses reporting beneficial effects of ecological intensive management (organic farming) on soil organic carbon (SOC) stocks^9,10^. In the respective meta-analyses, the beneficial effect of ecological intensive management was attributed to higher organic C inputs and distinct plant traits. Plants can influence microbial community composition^74^, for example *via* litter quality^75^ and rhizodeposition^76^. Measurements on six sites across Europe (including the ones assessed in the current study) showed that higher crop residue decomposability in conventional intensive systems explain the beneficial effect of ecological intensive systems on SOC^10^. However, the beneficial effect of ecological intensification on SOC was absent if litter N concentrations of the compared systems were not distinct enough. Among the sites with no beneficial effect of ecological intensification^10^ on SOC are the sites used in the current experiment. Linear regression between soil properties, litter and vegetation diversity and composition with the sub-part of the proteolytic microbial community composition under the influence of management (coordinates of the first axis of the db-RDA) was used to identify possible drivers for the observed management effect. We could not identify soil properties to correlate with proteolytic microbial community composition (SI Table 4), but there was a positive correlation between litter properties and proteolytic (*apr*) microbial community composition (Table 4). In detail, litter N properties were not correlated with proteolytic (*apr*) microbial community composition, but litter P properties were. Out of all litter- P properties associated with proteolytic (*apr*) microbial community composition, exclusively litter N:P ratios were not conditioned by country (Table 4). The lower litter N:P ratios in the ecological intensively *vs*. conventional intensively managed systems were mostly due to higher litter P concentration. Lower abundance of grasses and legumes relative to forbs in ecological intensive plots (except in Switzerland, Table 1 and Table 4) can in part explains lower litter N:P ratios^77,78^. Our results indicate that the management effect on proteolytic microbial community composition might act through contrasting litter qualities in ecological intensive and conventional intensive systems. Lower litter N:P ratios in plant communities on ecological intensively managed plots probably release more P and less N^79^. Such differences could have modified nutritional constraints for microbes and explain the observed selection of different proteolytic microbial communities. Under the respective condition, microbial communities might have developed a more efficient enzymatic machinery and can thus better extract N from soil organic matter, some of which being profitable to forage plants. Lower litter N:P ratios might have also potentially selected for more copiotrophic microbes^80^ and thus increase the mineralization potential. Characterized by a low biomass N:P ratio and fast growth rates^81^, copiotrophic microbes could shift from N immobilization to N mineralization at a lower N:P ratio compared to oligotrophic microbes^79^. P additions can select for microbial community with traits associated to a more copiotrophic lifestyle resulting in increased N mineralization and soil inorganic N concentration and, paralleled by decreased organic N concentration^82^. Thus, differences in litter P concentration and its repercussion on litter N:P might modify proteolytic microbial community composition translating into a positive effect on forage-N uptake. These results encourage further experimental research on how litter can shape soil (proteolytic) microbial communities and affect their functioning.

### Rain regime affects N-related ecosystem processes and -soil indicators but only marginally -microbial communities

Consistent rain regime effects on N-related ecosystem processes, and soil indicators were identified across countries (Table 2). In general, forage-N uptake increased under wet and decreased under dry rain regime (Fig. 3, SI Fig. 3). N-related soil indicator responses to rain regime were in line with a current meta-analysis^22^ with increase of NH_4_^+^ under reduced precipitation, while no effect on NO_3_^−^ was present. However, in the wet rain regime, soil NO_3_^−^ + NO_2_^−^ decreased, probably due to enhanced losses *via* leaching, plant uptake or gaseous emissions^83^.

Microbial communities are also supposed to be sensitive to soil water potential and thus expected to be responsive to rain regimes. A meta-analysis identified microbial activity to decrease with decreasing soil moisture in a consistent way across biomes and climatic conditions^84^. Despite the strong rain regime effects on N-related ecosystem processes and soil indicators, only N-related microbial activity was significantly affected by rain regimes whereas no effects on N-related microbial community abundance, diversity and composition were observed (Tables 2 and 3). This response confirms that rain regime does affect N-related microbial communities but suggests that activity is more water sensitive than the other microbial parameters. Furthermore, rain regime effects on N-related microbial activity did not follow a rain gradient as hypothesized. Conversely, we observed that all rain regimes tend to decrease activity, which suggests that different mechanisms are acting in these contrasted rain regimes but all leading to a decrease in activity (Piton & al. under review). Studies assessing effects of soil moisture on N-related functional genes are scarce, making comparisons with our results difficult.

Several explanations are possible for the weak rain regime effect on N-related microbial communities observed in the current experiment, while higher climate sensitivity has been observed for the same functional genes (*apr* and *npr*)^36^. The fact that the dry, wet and intermittent rain regimes were distinct enough to affect plant growth and N uptake but not enough to affect N-related microbial communities, potentially indicates lower sensitivity of N-related microbial communities to soil moisture variation compared to plants. All TMEs, even the dry ones, received water during the 263 days they experienced distinct rain regimes (amount and intensity differed) and current microbial community characterization was conducted only on the top soil layer (5 cm). Thus, although soil moisture was quite distinct at the sampling time, the microbial community might have been able to profit from the few precipitation events during the dry rain regime whereas plants did not^85^. Lastly, rain regime effects on N-related microbial communities might have been compensated by other factors such as enhanced rhizodeposition by plants under moderate drought^86^.

### Limitation of the experimental approach and perspectives

The use of TMEs, which simulate processes and interactions in a portion of the terrestrial ecosystem, is much closer to ecological realism than the more controlled laboratory experiments based on disturbed soil samples. However, our experimental conditions differed from original field sites and thus might have influenced our results, which calls for cautious interpretation. First, the same steady incubation temperature was used for all TMEs, whereas climatic conditions differed between original sites. This experimental design was chosen to study rain regime effects independent of temperature. However, this probably caused stronger effects on soil from the relatively cold site (French mountains) and underestimated effects on soil from the relatively warm site (Portugal). Moreover, modifications of rain regimes are likely associated with global climate change variables such as temperature and atmospheric greenhouses gas concentrations with potent direct, indirect and/or combined effects on soil microorganisms and functioning^62,87^. Another limitation of the current study is the absence of fertilization in the TMEs, except from litter residue application at the start of the incubation, which might have reduced forage-N uptake and NO_3_^−^ leaching compared to field conditions. This effect might be more pronounced in the most fertilized sites (Table 1). Another factor likely leading to an underestimation of overall plant growth and forage-N uptake, is the number of plant harvests, which was more frequent than in field conditions. This might be especially important for the Swiss TMEs, as the field site is usually mowed only four to five times a year. In relation to the discussed limitations we propose similar experiments using sites with less pronounced differences between managements and/or using more sites across Europe. As our understanding of N-related microbial processes under simulated climate change increases, so will our ability to test the obtained insights under more realistic field conditions. Furthermore, part of the variation of N-related microbial community properties and N-cycling processes was not explained by our models (Tables 2,4 and SI Table 3), demonstrating that important factors controlling proteolytic microbial communities and ecosystem functioning were not included in our study. For that reason, investigations into the associations between ecosystem processes and other functional groups than considered in the present study are strongly encouraged. Furthermore, we emphasize to move from TME incubation studies to *in situ* experiments using rainfall manipulation (for example with rain shelters^88^).

### Synthesis

Our experiment revealed strong direct impacts of rain regimes on N-cycling processes, whereas N-related microbial communities were exclusively affected in terms of activity. We did not find management to directly affect N-related microbial community abundance, diversity and activity and abiotic N-related soil indicators. However, management did affect N-related microbial community composition which appeared to be linked with forage-N uptake and NO_3_^−^ leaching.

Furthermore, we found SOM concentration to affect N-related microbial communities resulting in increased forage-N uptake and decreased NO_3_^−^ leaching (Fig. 3, SI Figure 4). The SOM effect was independent of management suggesting that management effects on microbial community act through other paths than SOM concentration. Our additional analyses indicate a role of litter P associated properties, especially litter N:P ratio, in shaping N-related microbial community composition (Table 4). While the importance of litter traits for soil C sequestration has been shown before^10^, our results also suggest a role of plant functional characteristics on the N-cycle through the proteolytic microbial community.

## Supplementary information

Supplementary Information.

Supplementary Information.
