# Supplementary material for: Compared to conventional, ecological intensive management promotes beneficial proteolytic soil microbial communities for agro-ecosystem functioning under climate change-induced rain regimes
Source: Sci Rep. 2020 Apr 29;10:7296. doi: 10.1038/s41598-020-64279-8 (PMC7190635; doi:10.1038/s41598-020-64279-8)
Supplement: Supplementary file 1 — Supplementary Information. [file 41598_2020_64279_MOESM1_ESM.docx]

**Compared to conventional, ecological intensive management promotes beneficial proteolytic soil microbial communities for agro-ecosystem functioning under climate change-induced rain regimes**

Martina Lori^1,2+,^ Gabin Piton^3+*^, Sarah Symanczik^1^, Nicolas Legay^4,^, Lijbert Brussaard^5^, Sebastian Jaenicke^6^, Eduardo Nascimento^7^, Filipa Reis^7^, Paulo Sousa^7^, Paul Mäder^1^, Andreas Gattinger^1,2^, Jean-Christophe Clément^3,8^, Arnaud Foulquier^3^

+ Shared first co-authorship

* corresponding author: gabinpiton@gmail.com

^1^ Department of Soil Sciences, Research Institute of Organic Agriculture (FiBL), Ackerstrasse 113, 5070 Frick, Switzerland

^2^ Organic Farming with focus on Sustainable Soil Use, Karl-Glöckner-Str. 21 C, Justus-Liebig University Giessen, 35394 Giessen, Germany

^3^ Univ. Grenoble Alpes, Univ. Savoie Mont Blanc, CNRS, LECA, 38000 Grenoble, France

^4^ École de la Nature et du Paysage, INSA Centre Val de Loire, 41000 Blois - CNRS, CITERES, UMR 7324, 37200 Tours, France.

^5^ Soil Biology Group, Wageningen University & Research, P.O. Box 47, 6700 AA Wageningen, The Netherlands

^6^ Bioinformatics and Systems Biology, Heinrich-Buff-Ring 58, Justus-Liebig-University Giessen, 35392 Giessen, Germany

^7^ Centre for Functional Ecology, Department of Life Sciences, University of Coimbra, 3000-456 Coimbra, Portugal

^8^ Univ. Savoie Mont Blanc, INRAE, CARRTEL, 74200, Thonon-Les-Bains, France

# SI Figures

**SI Figure 1: Terrestrial model ecosystem (TME) sampling in the different countries and the joint experiment including all TMEs.** In autumn 2015, 96 TMEs (30 cm depth x 16.5 cm diameter) were extracted from three countries (France, Portugal, Switzerland) from four plots under conventional intensive farming and four plots under ecological intensive management within each country, with four TME extracted per plot. TME were extracted with stainless-steel-extraction tube and a hydraulic excavator and were subsequently transported to Coimbra (Portugal) for a joint experiment.

**SI Figure 2. Schematic overview of rain regimes.** Four differential rain regimes were applied to the terrestrial model ecosystems (TMEs) according to the soils’ maximum water holding capacities (WHCmax).

SI Figure 3: Forage-nitrogen (N) uptake, NO_3_^-^ leaching, soil organic matter (SOM), NH_4_^+^, NO_3_^-^ + NO_2_ and dissolved organic nitrogen (DON) as affected by rain regime. Results derive from post hoc comparison on mixed effect models. Different letters indicate significant differences between treatments according to Tukey’s honest significant difference test with a significance level of p≤0.05. Error bars represent standard error, n=24


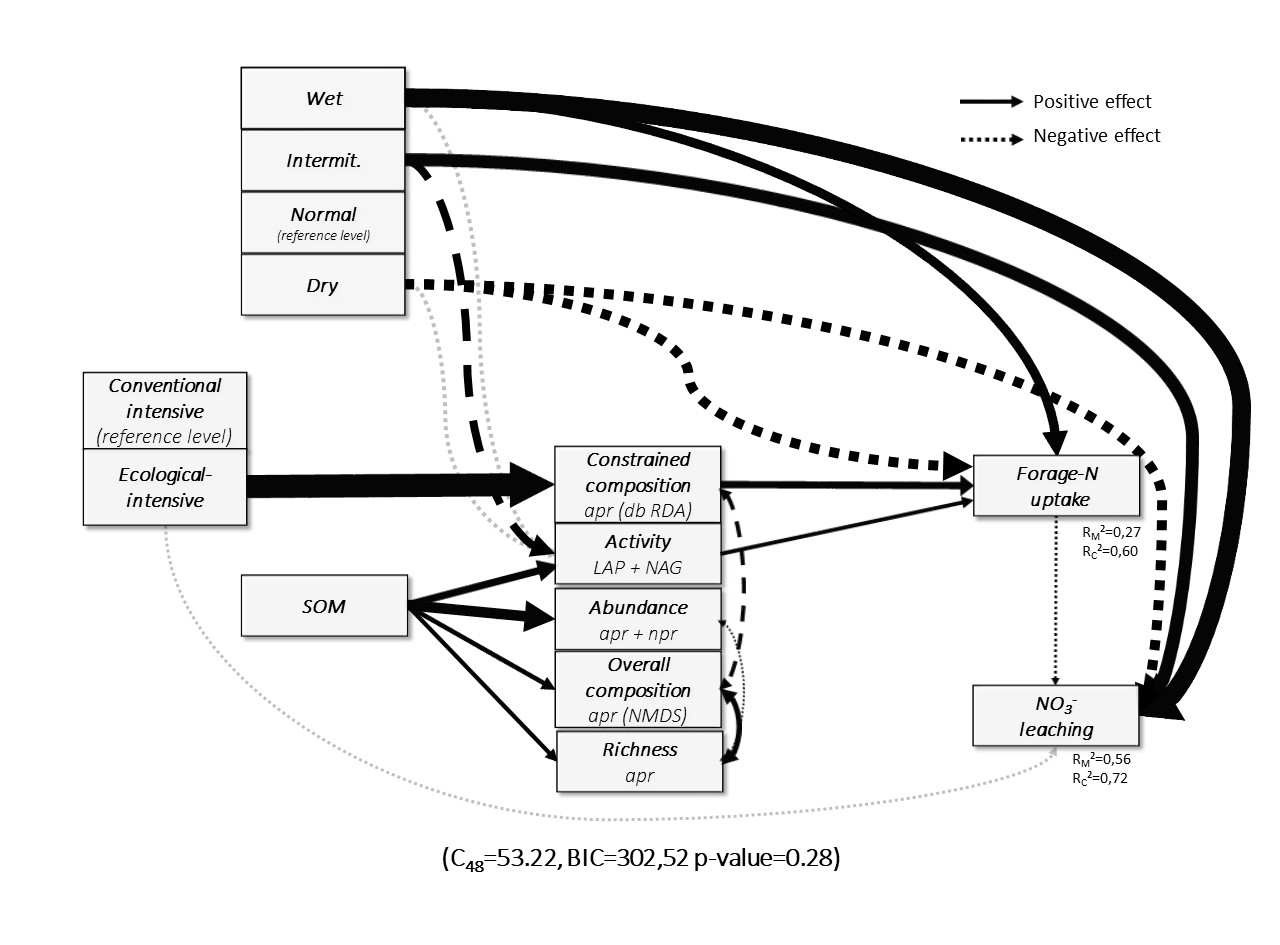


**SI Figure 4: Full structural equation model (SEM) representing paths from rain regime and management to nitrogen (N)-cycling processes through soil organic matter (SOM) concentration and N-related microbial communities.** Arrow width represents standardized effect size, black arrows represent significant paths, light grey arrows represent non-significant paths conserved during model selection process (see SI Table 3 for all coefficient values and significance). Marginal R² (R²_m_) and conditional R² (R²_c_) are given only for ecosystem processes (see SI Table 3 for R² of all endogenous variables). One-headed arrows represent causal relationships while double-headed arrows represent free correlations. LAP= leucine aminopeptidase, NAG= β-1,4-N-acetylglucosaminidase, *apr*= *alkaline metallopeptidase, npr*= *neutral metallopeptidase,* NMDS= Non-metric multidimensional scaling, db-RDA= distance based redundancy analysis*.* ‘Constrained composition *apr* (db-RDA)’= projected score of the first db-RDA axis of proteolytic *(apr)* microbial community composition (representing the sub part of the composition constrained by management), 'Overall composition *apr* (NMDS)’= projected score of the first NMDS axis of proteolytic *(apr)* microbial community (representing the overall proteolytic microbial community composition).

# SI Tables

**SI Table 1: Overview of oligonucleotide sequences and cycling conditions used for quantitative polymerase chain reaction (qPCR) and amplicon sequencing**. *apr= alkaline metallopeptidase, npr= neutral metallopeptidase,* FP= Forward Primer, RP= Reverse Primer, C1= Fluidigm-tag C1, C2= Fluidigm-tag C2

| **Name** | **Target gene** | **C1/C2 Tag** | **Sequence** | **Reference** | **Annealing temperature [° C]** | **Number**  **of Cycles** | **Primer concentration** |
| --- | --- | --- | --- | --- | --- | --- | --- |
| FP aprI | *apr* |  | TAY GGB TTC AAY TCC AAY AC | Bach et al. (2001) | 55 | 35 | 750nM |
| RP aprII | *apr* |  | VGC GAT SGA MAC RTT RCC | Bach et al. (2001) |  |  | 750nM |
| FP nprI | *npr* |  | GTD GAY GCH CAY TAY TAY GC | Bach et al. (2001) | 55 | 35 | 750nM |
| RP nprII | *npr* |  | ACM GCA TGB GTY ADY TCA TG | Bach et al. (2001) |  |  | 750nM |
| C1-FP aprI | *apr* | ACACTGACGACATGGTTCTACA | TAY GGB TTC AAY TCC AAY AC | Lori et al. (2018) | 54 | 39 | 600nM |
| C2-RP aprII | *apr* | TACGGTAGCAGAGACTTGGTCT | VGC GAT SGA MAC RTT RCC | Lori et al. (2018) |  |  | 600nM |

**SI Table 2. Effects of rain regime and management on proteolytic microbial community composition country-wise.** Effects were assessed by PERMANOVA (999 permutations) on a distance matrix based on *alkaline metallopeptidase* (*apr*) operational taxonomic units (OTUs) using Bray-Curtis distance metrics to assess treatment effect per countries. Df= degree of freedom, n.s= non-significant. Sample 75 and 27 of the Portuguese sub dataset were identified as outliers and got removed.

| Country | Effects | Df | F | R^2^ | p |
| --- | --- | --- | --- | --- | --- |
| France | Management | 1 | 1.74 | 0.05 | **0.001** |
|  | Rain regime | 3 | 0.91 | 0.09 | n.s |
|  | Management x Rain regime | 3 | 0.90 | 0.09 | n.s |
|  |  |  |  |  |  |
| Switzerland | Management | 1 | 1.19 | 0.04 | 0.014 |
|  | Rain regime | 3 | 1.03 | 0.10 | n.s |
|  | Management x Rain regime | 3 | 0.97 | 0.10 | n.s |
|  |  |  |  |  |  |
| Portugal | Management | 1 | 1.23 | 0.04 | **0.008** |
|  | Rain regime | 3 | 0.93 | 0.10 | n.s |
|  | Management x Rain regime | 3 | 0.92 | 0.10 | n.s |
|  |  |  |  |  |  |

# SI Table 3. Data underlying structural equation model depicted in Figure 5 representing rain regime and management effects on nitrogen (N)-cycling processes through soil organic matter (SOM) concentration and N-related microbial communities. Estimate= estimated standardized coefficient, p= p-value, bold parameters are present in both full (SI Figure 4) and simplified (Figure 3) SEM. NMDS= Non-metric multidimensional scaling, db-RDA= distance based redundancy analyses, *apr= alkaline metallopeptidase*, *npr= neutral metallopeptidase,* LAP= leucine aminopeptidase extracellular enzyme activities, NAG= β-1,4-N-acetylglucosaminidase. Marginal R² (R²_m_) and conditional R² (R²_c_) for endogenous variables are: Overall composition (*apr*): R²_m_= 0.09, R²_c_= 0.72, constrained composition (*apr*): R²_m_= 0.80, R²_c_=0.88, richness (*apr*): R²_m_= 0.10, R²_c_=0.32, activity (LAP + NAG): R²_m_= 0.36, R²_c_= 0.39, abundance (*apr + npr*): R²_m_= 0.56, R²_c_= 0.61, forage-N uptake: R²_m_= 0.27, R²_c_= 0.60, NO_3_^-^ leaching: R²_m_= 0.56, R²_c_= 0.72.

| **Response** | **Predictor** | **Estimate** | **p** |
| --- | --- | --- | --- |
| **Hypothesized causal relationship** |  |  |  |
| Overall structure (*apr*, NMDS Axis 1) | SOM | 0.332 | 0.018 |
| **Constrained structure (*apr*, db-RDA Axis 1)** | **Management (Ecological intensive)** | **1.800** | **0.010** |
| Richness (*apr*) | SOM | 0.313 | 0.020 |
| **Activity (LAP + NAG)** | **Rain regime (Dry)** | **-0.305** | **0.190** |
| **Activity (LAP + NAG)** | **Rain regime (Flood)** | **-0.312** | **0.179** |
| **Activity (LAP + NAG)** | **Rain regime (Intermittent)** | **-0.638** | **0.007** |
| **Activity (LAP + NAG)** | **SOM** | **0.548** | **p<0.001** |
| **Activity (LAP + NAG)** | **Management (Ecological intensive)** | **0.224** | **0.320** |
| Abundance (*apr* + *npr*) | SOM | 0.756 | p<0.001 |
| **Forage-N uptake** | **Rain regime (Dry)** | **-0.734** | **0.004** |
| **Forage-N uptake** | **Rain regime (Flood)** | **0.630** | **0.010** |
| **Forage-N uptake** | **Rain regime (Intermittent)** | **-0.022** | **0.929** |
| **Forage-N uptake** | **Constrained structure (*apr*, db-RDA Axis 1)** | **0.395** | **0.035** |
| **Forage-N uptake** | **Activity (LAP + NAG)** | **0.228** | **0.048** |
| **NO_3_^-^ leaching** | **Rain regime (Dry)** | **-0.727** | **p<0.001** |
| **NO_3_^-^ leaching** | **Rain regime (Flood)** | **1.426** | **p<0.001** |
| **NO_3_^-^ leaching** | **Rain regime (Intermittent)** | **0.982** | **p<0.001** |
| **NO_3_^-^ leaching** | **SOM** | **-0.077** | **0.505** |
| **NO_3_^-^ leaching** | **Forage-N uptake** | **-0.206** | **0.006** |
| **NO_3_^-^ leaching** | **Management (Ecological intensive)** | **-0.217** | **0.225** |
| **Free covariation** |  |  |  |
| Abundance (*apr + npr*) | Richness (*apr*) | 0.145 | 0.084 |
| **Constrained structure (*apr*, db-RDA Axis 1)** | **Activity (LAP + NAG)** | **-0.020** | **0.423** |
| Overall structure (*apr*, NMDS Axis 1) | Richness (*apr*) | 0.384 | p<0.001 |
| Overall structure (*apr*, NMDS Axis 1) | Constrained structure (*apr*, db-RDA Axis 1) | -0.299 | 0.002 |

**SI Table 4. Management effect on soil properties and their correlation with proteolytic (*apr*) microbial community composition assessed using mixed effect model with country as random factor.** R²_m_= marginal R², R²_c_= conditional R²_c._ C= carbon, N= nitrogen, P= phosphorus, SOM= soil organic matter, DOC= dissolved organic carbon, DON= dissolved organic nitrogen, DMN= dissolved mineral nitrogen, db-RDA= distance based redundancy analyses, *apr*= *alkaline metallopeptidase*.

| **Soil properties** |  | **Management effect** | | |  | **Correlation with constrained *apr* composition (db-RDA axis 1)** | | |
| --- | --- | --- | --- | --- | --- | --- | --- | --- |
|  |  | **p** | **R²_m_** | **R²_c_** |  | **p** | **R²_m_** | **R²_c_** |
|  |  |  |  |  |  |  |  |  |
| SOM (%) |  | 0.46 | 0.00 | 0.82 |  | 0.70 | 0.01 | 0.01 |
| C (%) |  | 0.91 | 0.00 | 0.72 |  | 0.99 | 0.00 | 0.00 |
| N (%) |  | 0.43 | 0.01 | 0.73 |  | 0.48 | 0.02 | 0.02 |
| P (mg/Kg) |  | 0.35 | 0.03 | 0.17 |  | 0.07 | 0.14 | 0.14 |
| C:N |  | 0.41 | 0.03 | 0.14 |  | 0.25 | 0.06 | 0.06 |
| C:P |  | 0.89 | 0.00 | 0.49 |  | 0.37 | 0.03 | 0.03 |
| N:P |  | 0.50 | 0.01 | 0.52 |  | 0.12 | 0.10 | 0.10 |
| pH |  | 0.65 | 0.01 | 0.31 |  | 0.63 | 0.01 | 0.01 |
| DOC (mgC/Kg) |  | 0.35 | 0.01 | 0.66 |  | 0.68 | 0.01 | 0.01 |
| NH_4_^+^ (mgN/Kg) |  | 0.71 | 0.01 | 0.16 |  | 0.62 | 0.01 | 0.01 |
| NO_3_^-^+ NO_2_^-^(mgN/Kg) |  | 0.62 | 0.01 | 0.54 |  | 0.36 | 0.04 | 0.04 |
| DON (mgN/Kg) |  | 0.95 | 0.00 | 0.00 |  | 0.35 | 0.04 | 0.04 |
| PO_4_ (µgP/g dry soil) |  | 0.48 | 0.02 | 0.08 |  | 0.16 | 0.08 | 0.08 |
| DOC:DMN |  | 0.26 | 0.04 | 0.25 |  | 0.40 | 0.03 | 0.03 |
| DMN:PO_4_ |  | 0.25 | 0.05 | 0.22 |  | 0.08 | 0.13 | 0.13 |
| DOC:PO_4_ |  | 0.26 | 0.05 | 0.11 |  | 0.28 | 0.05 | 0.05 |
| Silt (%) |  | 0.581 | 0.00 | 0.95 |  | 0.657 | 0.01 | 0.05 |
| Clay (%) |  | 0.884 | 0.00 | 0.13 |  | 0.668 | 0.01 | 0.01 |
| Sand (%) |  | 0.707 | 0.00 | 0.93 |  | 0.629 | 0.02 | 0.06 |
